# Clinical Efficacy of Platelet-Rich Plasma in the Treatment of Neurotrophic Corneal Ulcer

**DOI:** 10.1155/2018/3538764

**Published:** 2018-06-20

**Authors:** Dominika Wróbel-Dudzińska, Jorge Alio, Alejandra Rodriguez, Ewa Suchodoła-Ratajewicz, Ewa Kosior-Jarecka, Beata Rymgayłło-Jankowska, Agnieszka Ćwiklińska-Haszcz, Tomasz Żarnowski

**Affiliations:** ^1^Department of Diagnostics and Microsurgery of Glaucoma, Medical University of Lublin, Lublin, Poland; ^2^Department of Cornea and Refractive Surgery, Vissum Corporation, Alicante, Spain; ^3^Research and Development Department, Vissum Corporation, Alicante, Spain; ^4^Department of General Ophthalmology, Medical University of Lublin, Lublin, Poland

## Abstract

**Purpose:**

Platelet-rich plasma (PRP) is an autologous blood product without preservatives and rich in proteins and growth factors which make it possible for cells to differentiate, proliferate, and migrate, thus stimulating healing and regeneration of tissues. The aim of this study was to evaluate the efficiency of autologous platelet-rich plasma in the treatment of neurotrophic keratopathy.

**Methods:**

The study group consists of 25 patients with nonhealing corneal ulcers due to herpes simplex or herpes zoster infection and facial nerve or trigeminal nerve paralysis as a result of a neurosurgical operation caused by a tumour or stroke. The patients were given autologous platelet-rich plasma drops five times a day and additionally preservative-free artificial tears and a vitamin A ointment at night for maximum 3 months. The following were evaluated: best corrected visual acuity (BCVA), healing of corneal surface, subjective symptoms, and changes in corneal thickness with the use of anterior segment optical coherent tomography (AS-OCT).

**Results:**

BCVA before the treatment was 0.10 ± 0.14, and after the treatment it was –0.3 ± 0.27 (*p*=0.001). Improved visual acuity and less subjective symptoms were observed in all patients. Complete healing of the ulceration was observed in 20 patients (80%). Four patients (16%) experienced considerable improvement of their clinical condition (reduced size and depth of the ulceration and inflammatory state: smaller conjunctival injection and swelling, improved visual acuity, and less subjective symptoms). In one of the patients, an amniotic membrane was transplanted due to the lack of improvement of his local condition. In all patients, the progression of corneal thinning was stopped. An average corneal thickness in its thinnest point was 322.3 ± 125.8 *µ*m before the treatment, and 404.5 ± 118.7 *µ*m (*p* < 0.05) after the treatment. None of the patients reported general or local side effects of the treatment.

**Conclusions:**

Autologous platelet-rich plasma is a blood-based product which seems efficient in the treatment of neurotrophic keratopathy.

## 1. Introduction

Neurotrophic keratitis (NK) is a rare degenerative disease characterized by reduction or absence of corneal sensitivity and consequently dysfunction of corneal healing process (reduction in the lacrimation reflex, corneal epithelium disruption, healing impairment, development of corneal opacification and stromal ulcers, and melting and perforation), followed by irreversible visual deficit [1, 2]. The most common causes of neurotrophic keratitis are presented in Table 1 [3, 4].

Diagnosis of NK is challenging, and the success may be achieved by acquired extremely detailed medical history to consider all potential risk factors and conducted comprehensive examination of the ocular surface. Unfortunately, most often NK is diagnosed in its advanced stages when visual acuity has worsened as a result of damage to the corneal epithelium and corneal scarring. Patients rarely complain about other ocular symptoms such as red eye, pain, discomfort, burning sensation, and the foreign body sensation, since their corneal sensory innervation has been impaired. Individuals whose palpebral fissure does not close completely complain about watery eyes. It is very important that any diagnostic procedures should include taking a complete medical history into account general diseases coexistent with NK as well as any previous surgical procedures. The NK was classified as an orphan disease and divided into three stages according to the Mackie classification (Table 2) [5].

In the case of neurotrophic keratopathy, both diagnostic process and treatment are extremely difficult. There is no one efficient course of treatment whose aim would be to improve the condition of corneal epithelium and at the same time to prevent the development of corneal ulcers and their subsequent perforation. Taking into consideration pathophysiology of the disease, the role of neurotransmitters and growth factors, cytokines, a search for agents which would regenerate damaged epithelium continues. In the light of the enormous interest in regenerative medicine, blood-based products have started to receive more attention. One of them is platelet-rich plasma (PRP), commonly used in many areas of medicine such as oral and maxillofacial surgery, plastic and reconstructive surgery, orthopaedics, cardiovascular surgery, and esthetic medicine. Therapy of NK is based on promoting corneal healing; thus, such grate reservoir of growth factors and cytokines seems to be desirable option. The purpose of this work was to evaluate the efficiency and safety of using autologous platelet-rich plasma in the treatment of neurotrophic keratopathy.

## 2. Material and Methods

This is a prospective, nonrandomized, observational consecutive pilot study that followed the tenets of the Declaration of Helsinki. After approval by the local Ethical Committee, informed consent of the patients was obtained before the initiation of the study and patient recruitment.

We studied 25 subjects (14 females and 11 males) recruited from the Department of Diagnostic and Microsurgery of Glaucoma, University of Lublin, Poland, from January 2016 to September 2017.

### 2.1. Inclusion Criteria

Patients older than 18 years with neurotrophic corneal ulcers due to herpes infection or cranial nerve V or VII palsy in the stages II or III according to the Mackie classification, not responding to conventional treatment, were recruited.

All patients underwent a comprehensive ophthalmological examination and responded to the survey questionnaire, which concerned subjective symptoms such as photophobia, lacrimation, pain, and discomfort (*Questions* required either *Yes*/*No* response). Microbiological examination of corneal scrape was performed to exclude bacterial and fungal infection.

### 2.2. Exclusion Criteria

Patients with a history of corneal surgery, trauma, chemical burns, contact lens abuse, topical anesthetic abuse, corneal dystrophies, or neurotrophic corneal ulcers stages I according to the Mackie classification were excluded.

### 2.3. Treatment

The patients were treated with eye platelet-rich plasma drops five times a day, preservative-free artificial tears five times a day, and vitamin A ointment once at night for maximum 3 months. Some patients with NK due to herpes simplex or herpes zoster infection received additional general (orally acyclovir 400 mg four times a day) and/or topical antiviral treatment (with acyclovir 5 times a day). Those with infectious keratitis with/without hypopyon were given topical antibiotics treatment (with tobramycin and ciprofloxacin four times a day), cycloplegics treatment (with atropine once a day), and a treatment with the use of a bandage contact lens.

### 2.4. Patient Follow-Up

Patients attended follow-up consultations on days 1, 7, 14, and 21 and at 1, 2, and 3 months after the treatment initiation. The treatment was stopped after 3 months or when the corneal healing was achieved.

### 2.5. Main Outcome Measures

The following were assessed: best corrected visual acuity (BCVA) for the distance measured on the Snellen chart (decimal scale); anterior segment evaluation, healing of the corneal surface (size of the epithelial defect/ulcer: width and height measured by calipers) with fluorescein staining; corneal sensitivity in the central and peripheral cornea measured by cotton wisp test; condition of the conjunctiva (oedema and hyperemia) and the presence of any pathological discharge; subjective symptoms (pain, discomfort, lacrimation, photophobia, and foreign body sensation) given by the patients; and the results of the anterior segment optical coherence tomography (AS-OCT) (size and depth of the epithelial defect/ulcer, corneal thickness at the thinnest location) conducted with a Casia I (Tomey) photographic assessment.

In the ophthalmologic examination, special attention was paid to the size and depth of the epithelial defect and ulceration. The size (width and height) of the corneal epithelial defect was measured by calipers and classified as large when it exceeded 4.0 mm, medium when it was between 2.0 and 4.0 mm, and small when it was smaller or equal to 2.0 mm.

Subjective symptoms were evaluated based on the self-administered questionnaire, which was completed once at the time of enrollment in the study (baseline) and again after treatment with eye platelet-rich plasma (E-PRP). Patients were asked to report any change in their perception of pain, discomfort, foreign body sensation, photophobia, and lacrimation, giving yes or no answer.

### 2.6. Autologous PRP Preparation

Platelet-rich plasma drops were prepared according to the previous description [6, 7]. The strict sterility conditions using sterile and disposable materials and operating inside a laminar flow hood were needed to prepare PRP. Autologous drops were produced from 40 to 80 ml of whole blood, obtained from patients by venipuncture and collected into 10 ml sterile tubes containing 1 ml of sodium citrate. The tubes including blood were spun at room temperature 1400 rpm for 10 minutes. 90% of obtained plasma was used as the final product, and then three to four millilitres were placed into new, sterilized 10 ml bottles with eye drop applicators. Patients were given the instruction to wash their hands prior to the application of the product, to keep the area of application clean and not to touch the eyedropper. The bottle in use should be kept at +4°C and the rest at –20°C.

### 2.7. Statistical Analysis

Statistical analysis was conducted using Excel and Statistica 12 programs. The Shapiro–Wilk test was used to test the normality of the distribution of the evaluated qualities. The value of *p* < 0.05 was considered to be statistically significant. Depending on the level of measurement of variables, different statistical tests and coefficients were employed: *t*-Student and ANOVA.

## 3. Results

The patients' characteristics are shown in Table 3. Average patient age was 66.08 ± 16.45. In 15 patients, the persistence epithelial defect or ulceration was caused by a herpes zoster or simplex infection, and in 10 patients it was the result of an injury to the trigeminal nerve and/or facial nerve incurred during a neurosurgical procedure. Eight patients presented with inadequate closing of the palpebral fissure as well as weakened Bell's reflex.

Improved visual acuity was observed in almost all the patients. Best corrected visual acuity before the treatment was 0.11 ± 0.14, and after the treatment it considerably improved to 0.3 ± 0.27 (*p*=0.001).

Mean time of the treatment was 9.2 ± 2.85 weeks. An average size of epithelial defect/ulceration before the treatment was 3.7 ± 1.1 x 2.8 ± 0.9 mm. After the treatment, complete reepithelialization of the persistence epithelial defect or ulcer was observed in 20 patients (80%), and 4 patients (16%) experienced considerable improvement, defined as at least 0.5 mm changes in the area of the epithelial defect/ulcer. Moreover, in almost all patients, reduction of the inflammatory state was observed: smaller conjunctival injection and swelling. Two of the participants had hypopyon, which disappeared after basic and additional treatment. The lack of corneal sensitivity was observed in 19 patients, the rest had it weakened. The corneal sensation did not change during the treatment. Unfortunately, in one of the patients an amniotic membrane was transplanted due to the lack of improvement of the local condition and high risk of corneal perforation.

A change in subjective symptoms was reported by the patients after the treatment. Lack of discomfort and photophobia was observed in 96% of participants. 6 patients declared (100%) reduction of foreign body sensation.

In all cases, the progression of corneal thinning was stopped. An average corneal thickness in its thinnest point was 322.3 ± 125.83 *µ*m before the treatment, and 404.53 ± 118.68 *µ*m (*p* < 0.05) after the treatment.

None of the patients reported general or local side effects of the treatment. Moreover, no recurrence of the corneal ulcer was observed within 10 months, which was our follow-up time.

**Figure 1:**
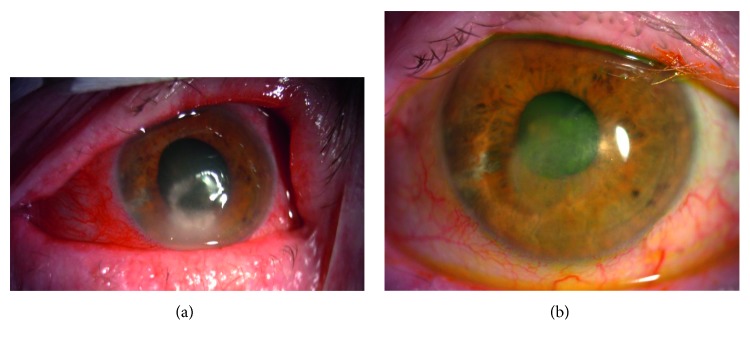
(a) Representative case of the patient (number 1) with stage 3 NK: large deep corneal ulcer with rolled edges of the epithelium, mix conjunctiva injection—hyperemia, stromal melting with hypopyon. (b) Picture of the patient number 1 after the treatment showing significant improvement: corneal scar.

**Figure 2:**
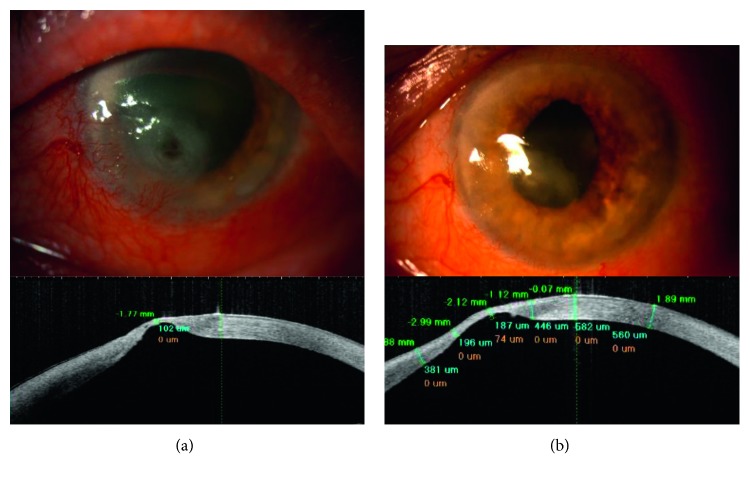
(a) Representative case of the patient (number 6) with stage 3 NK: large deep corneal ulcer, impending perforation, neovascularization, conjunctival, and iris hyperemia. The corneal thickness at the thinnest point was 102 *µ*m. (b) Clinical improvement of the patient number 6 after the treatment: large scar with superficial neovascularization. The corneal thickness improved to 187 *µ*m.

**Figure 3:**
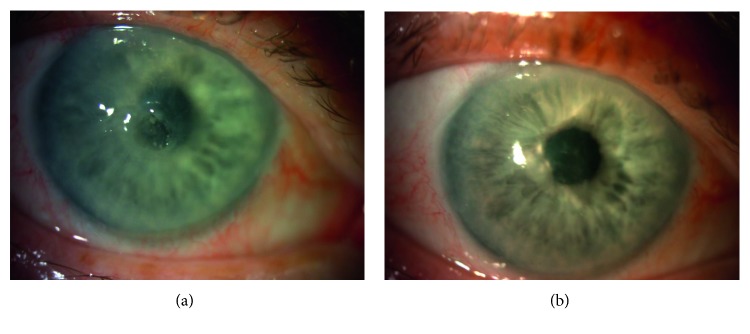
(a) Representative case of the patient (number 14) with stage 3 NK: small deep corneal ulcer with rolled edges of the epithelium, mix conjunctiva injection—hyperemia, stromal melting. (b) Clinical improvement of the patient number 14 after the treatment: complete healing of the ulcer with slight corneal haze.

Figures 1–3 show the clinical condition of patients, both before and after the treatment.

## 4. Discussion

Standard NK therapy depends on the stage in the Mackie classification [5]. The main target is to treat according to the etiopathogenesis and enhancing corneal healing to prevent corneal perforation. It is crucial to cure concomitant ocular disorders such as dry eye, exposure keratitis, or limbal stem cells deficiency. Usually, all topical medications which have been administered so far are stopped and the patient is given artificial tears without preservatives, and in severe cases in order to prevent secondary infection, topical antibiotics and bandage contact lenses are introduced [2, 8]. Topical glucocorticosteroids (GCs) are contraindicated in the case of a viral infection, as they increase viral replication, lengthen the time it takes for the stroma to heal and increase the risk of corneal perforation. However, they might prove useful when it comes to reducing inflammation. When treating NK, nonsteroidal anti-inflammatory drugs (NSAIDs) should be avoided, as well as eye drops with cyclosporine which has an adverse effect on the regeneration of corneal nerve fibres [2, 9].

To prevent melting and perforation of the cornea, some surgical procedures can be carried out, such as temporary or permanent tarsorrhaphy, injecting botulinum toxin A into the elevating muscle of upper eyelid, amniotic membrane or conjunctival patch graft, sympathectomy, transplanting mucuous membrane from the nose, Tutopatch with platelet-rich plasma, and transplanting a fragment of the sural nerve to restore corneal innervation [8, 10–13]. To improve the condition of epithelium and speed up the healing process, new unconventional methods of treatment have been introduced, including eye drops with autologous serum, umbilical cord serum, platelet-rich plasma, plasma rich in growth factors, neurotrophin, neuropeptides, and growth factors, for example, nerve growth factor (NGF), substance P (SP), and thymosin beta-4 [14–18].

According to the previous encouraging reports about promising therapeutic agent as matrix regeneration therapy in NK [17], Arvola et al. conducted their own study of neurotrophic ulcer treatment with RGTA®, where the outcomes were not so much satisfying from the efficacy point of view. They observed 2 patients with complete corneal healing, 4 patients with failure to heal, and in 1 case of perforation, no visual acuity improvement [19].

Currently, clinical trials are being conducted on the use of heparin sulphate and recombinant human nerve growth factor (rhNGF) in the treatment of NK [20, 21]. The group of scientists treated more than 45 eyes with moderate and severe neurotrophic ulcers with nerve growth factor eye drops observing healing in almost 100% of the cases [16]. Regrettably, genetically engineered NGF eye drops are still not available on the market in Poland.

The interest in blood hemoderivatives is constantly growing. According to Matsumoto's study, autologous serum helped heal epithelial disorders in 11 cases of NK. This clear acellular liquid fraction has properties comparable to natural tears as far as pH and osmolarity is concerned, thus this product is more effective in dry eye treatment. López-Plandolit et al. used plasma rich in growth factors for persistent corneal epithelial defects treatment reaching quite good results: epithelial defects healed in 17 of 20 cases. The main problem of this hemoderivative application is the preparation based on especially designed devices and double centrifugation technique with platelet activation [22].

In the light of the growing interest in blood-derived products and the lack of effective treatment for neurotrophic corneal ulcer, platelet-rich plasma seems very promising. It is an autologous product without any preservatives, extremely abundant in platelets, presenting a high concentration of cell adhesion molecules and growth factors. It is a known fact that an injury to the trigeminal nerve leads to the deficiency of neuropeptides, neurotransmitters, and growth factors maintaining homeostasis of ocular surface. That is why this mixture of growth factors and cytokines plays an essential role in regeneration process. It has been proven that platelet-rich plasma contains cytokines (e.g., PF4 and CD4OL) as well as growth factors such as PDGF (platelet-derived growth factor), TGF- (transforming growth factor-) ß1 and ß2, IGF- (insulin-like growth factor-) 1, VEGF, EGF, FGF-2, and IGF [13, 23]. Most of its efficiency PRP owes to PDGF factor which is the first growth factor to appear in a wound, stimulating revascularisation, collagen synthesis, and regeneration. Its role in healing process is to increase the number of repair cells, stimulate angiogenesis, support the development of new blood vessels, and activate macrophages responsible for cleaning the wound. TGF, secreted during platelet degranulation or actively produced by macrophages, acts as paracrine growth factor, in charge of chemotaxis and epithelium proliferation. Another important protein contained in alpha granules of platelets is EGF (endothelial growth factor). It speeds up corneal epithelium proliferation and has an antiapoptotic function. PF4 and CD4OL are proteins which control antibody-mediated immunity and cell-mediated immunity [24]. VEGF (vascular epithelial growth factor) and FGF- (fibroblast growth factor-) 2 take part in angiogenesis, as a result of which nutrients and progenitor cells are delivered to the wound by new vessels. Their mechanism is based on the function of blood platelets whose role is to prevent sudden blood loss as well as to repair vessel walls and surrounding tissue [25]. Interrupted endothelium is what activates blood platelets. Then, the activated alpha granules secrete numerous proteins and growth factors: cytokines; interleukins: IL-1*β*, IL-2, IL-4, IL-5, IL-6, IL-7, IL-8, IL-10, IL-12, IL-13, IL-15, IL-17, PF4, and CD4OL; growth factors: PDGF AA, AB, and BB; TGF-*β*1 i-*β*3; IGF-1; EGF; INF-*α* (interferon alpha); FGF; and fibronectins. As we know, most molecules are secreted in the first hour, then platelets synthesize and secrete cytokines and growth factors for another 7 days (it is related to the average life span of blood platelets which is 7 to 10 days) [26]. Growth factors bind to their transmembrane receptors in the damaged tissue, triggering a cascade of reactions: activation of endogenous signalling protein, expression of genes responsible for cell proliferation, formation of cell matrixes, and the synthesis of collagen. All these mediators set off the processes of cell differentiation, proliferation and migration, angiogenesis, and, by the same token, the process of regeneration of the damaged tissue. Furthermore, platelets secrete antibacterial proteins which have an antibiotic effect [27]. Since many questions concerning the detailed mechanism in which PRP works have still not been answered, further molecular study is necessary.

The results of the studies on the product's efficiency, both ours and the ones conducted by other researchers, are promising. Alio et al. used different varieties of platelet-rich plasma in 40 patients with nonhealing corneal ulcers. They observed complete healing of the ulceration in 23 patients, considerable improvement in 15 patients, and in 2 patients, the treatment did not bring any results. In majority of the cases, reduction of the inflammation and pain was observed. Moreover, visual acuity improved in about 60% of cases. The product was well tolerated and no adverse side effects were reported [6, 7].

The recurrent corneal erosions (RCE) treatment with platelet-rich plasma (PRP) eye drops was also evaluated. Lee et al. reported that the mean frequency of recurrence of corneal erosion was 0.06 ± 0.08 per month in the PRP eye drops treated group and 0.39 ± 0.24 per month in the conventional treatment group (*p*=0.003) [28].

The platelet-rich plasma is widely used in ophthalmology, other indications might be: macular hole and pterygium, after chemical burns [29–31].

## 5. Conclusion

Autologous platelet-rich plasma has been demonstrated to be effective in corneal neurotrophic ulcer treatment. The lack of preservatives, autologous quality, relative ease of its preparation, safety, and beneficial effects make PRP a promising therapeutic tool for future regenerative medicine. Despite the fact that recombinant synthetic products are available for neurotrophic ulcer treatment, those high-priced goods contain only a single growth factor.

## Figures and Tables

**Table 1:** Etiopathogenesis of neurotrophic keratitis.

Infectious factors	Herpes simplex
	Herpes zoster
	*Acanthamoeba*
	Leprosy

Corneal diseases	Chemical burns
	Foreign body
	Corneal dystrophies
	Refractive corneal surgery
	Corneal damage caused by contact lenses

Topical drugs	Chronic use of anesthetics, glucocorticoids, or *β*-blockers

Medicines used generally	Antihistamines
	Neuroleptics

V nerve palsy	Intracranial tumours
	Intracranial aneurysms
	Craniofacial injuries
	Neurosurgical procedures
	Stroke

Systemic diseases	Diabetes mellitus
	Multiple sclerosis
	Autoimmune diseases
	Sjögren syndrome
	Vitamin A deficiency

Other	Transscleral cyclophotocoagulation
	Pars plana vitrectomy with panretinal endophotocoagulation

Congenital	Riley–Day syndrome
	Möbius syndrome
	Goldenhar syndrome
	Ectodermal dysplasia
	Hereditary sensory neuropathy

**Table 2:** Clinical classification of neurotrophic keratopathy based on the Mackie classification.

Stage	Clinical findings
I	Corneal epithelial irregularity
	Gaule spots
	Superficial punctuate keratopathy
	Increase viscosity of tear mucus
	Decrease break-up time
	Superficial corneal neovascularization
	Stromal scarring
	Dellen

II	Persistent corneal epithelial defect with smooth and rolled epithelium edges
	Descemet's membrane folds
	Stroma swelling
	Anterior chamber inflammatory reaction (±hypopyon)

III	Corneal ulcer
	Stromal melting
	Corneal perforation

**Table 3:** Characteristics of the study group.

Patient number	Age	Gender	Etiology	Mackie classification	Ulcer size	Additional treatment	Additional surgical procedures	Corneal sensation
1	79	F	Herpes zoster	3	Big	Y	N	Lack
2	66	M	Herpes simplex	2	Big	Y	N	Weakened
3	60	F	Cranial nerve palsy	3	Big	Y	N	Lack
4	82	M	Cranial nerve palsy	2	Medium	N	N	Lack
5	89	M	Herpes simplex	3	Medium	Y	N	Lack
6	59	M	Cranial nerve palsy	3	Big	N	N	Lack
7	58	M	Herpes zoster	2	Big	Y	N	Lack
8	46	M	Cranial nerve palsy	2	Medium	N	N	Weakened
9	82	F	Herpes simplex	3	Big	Y	N	Lack
10	74	F	Cranial nerve palsy	2	Big	N	N	Weakened
11	43	F	Herpes simplex	3	Big	Y	Y	Lack
12	74	M	Cranial nerve palsy	3	Big	Y	N	Lack
13	83	M	Herpes simplex	3	Medium	Y	N	Lack
14	70	M	Herpes simplex	2	Small	Y	N	Weakened
15	80	M	Herpes simplex	2	Small	Y	N	Weakened
16	41	F	Cranial nerve palsy	2	Small	N	N	Lack
17	60	F	Herpes simplex	2	Small	Y	N	Lack
18	60	F	Cranial nerve palsy	3	Small	N	N	Lack
19	69	F	Cranial nerve palsy	2	Small	N	N	Lack
20	52	F	Herpes simplex	3	Medium	Y	N	Lack
21	74	F	Cranial nerve palsy	3	Medium	N	N	Lack
22	59	F	Cranial nerve palsy	3	Big	Y	N	Lack
23	81	F	Cranial nerve palsy	3	Big	Y	N	Lack
24	82	F	Herpes simplex	2	Medium	Y	N	Weakened
25	23	M	Cranial nerve palsy	2	Medium	N	N	Lack

F: female; M: male. Mackie classification stage 2: persistent epithelial defects with thickened and rolled edges, oedematous stroma, stage 3: corneal ulcer, stromal melting, impending perforation. Ulcer size (width and height): small: smaller or equal to 2 mm; medium: between 2 and 4 mm; big: greater than 4 mm. Y: yes; N: no.
